# P-1753. Impact of a Microbiology Reporting Change on Treatment Selection and Clinical Outcomes for Organisms at Low-Risk for Significant AmpC Production

**DOI:** 10.1093/ofid/ofae631.1916

**Published:** 2025-01-29

**Authors:** Ellie Hodapp, Patrick M Kinn, Bradley A Ford, Sarah Auerbach, Lukasz Weiner, Dilek Ince, Kelly M Percival

**Affiliations:** University of Iowa Health Care, Iowa City, Iowa; University of Iowa Hospitals & Clinics, Iowa City, Iowa; University of Iowa Hospitals and Clinics, Iowa City, Iowa; University of Iowa Hospitals and Clinics, Iowa City, Iowa; University of Iowa Hospitals & Clinics, Iowa City, Iowa; University of Iowa Hospitals & Clinics, Iowa City, Iowa; University of Iowa Health Care, Iowa City, Iowa

## Abstract

**Background:**

AmpC β-lactamase production can be induced after exposure to beta lactam antimicrobials, most notably ceftriaxone (CRO), leading to development of resistance on therapy. New guidance from IDSA indicates that the risk of AmpC enzyme production is not equal among all Enterobacterales and those deemed low-risk may be treated with CRO. The purpose of this study was to evaluate the impact of a microbiology reporting change made in collaboration with our antimicrobial stewardship program (ASP) to guide antibiotic selection for low-risk AmpC organisms.

**Figure:**
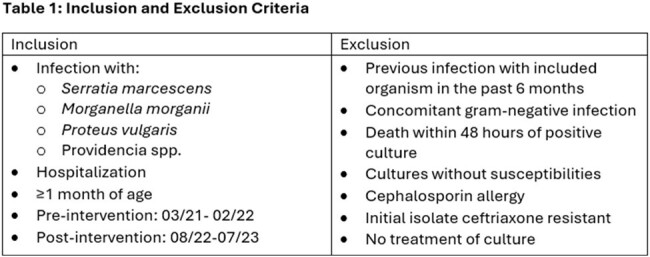

**Methods:**

This was a pre-test, post-test quasi-experimental study with data collected by chart review. A microbiology comment warning against the potential for inducible resistance on therapy with CRO was present pre-test and was removed in the post-test period. A didactic education session was given by ASP to medicine services. The study enrolled hospitalized patients ≥1 month old treated for infection with a low-risk AmpC organism (*Serratia marcescens*, *Morganella morganii*, *Proteus vulgaris*, and Providencia spp.) pre- (3/21-2/22) and post-intervention (8/22-7/23). Further inclusion and exclusion criteria are in **table 1**. The primary outcome was rate of carbapenem-sparing definitive treatment. Secondary outcomes were rate of CRO definitive therapy along with 30-day readmission, retreatment, mortality, and development of CRO resistance.

**Figure:**
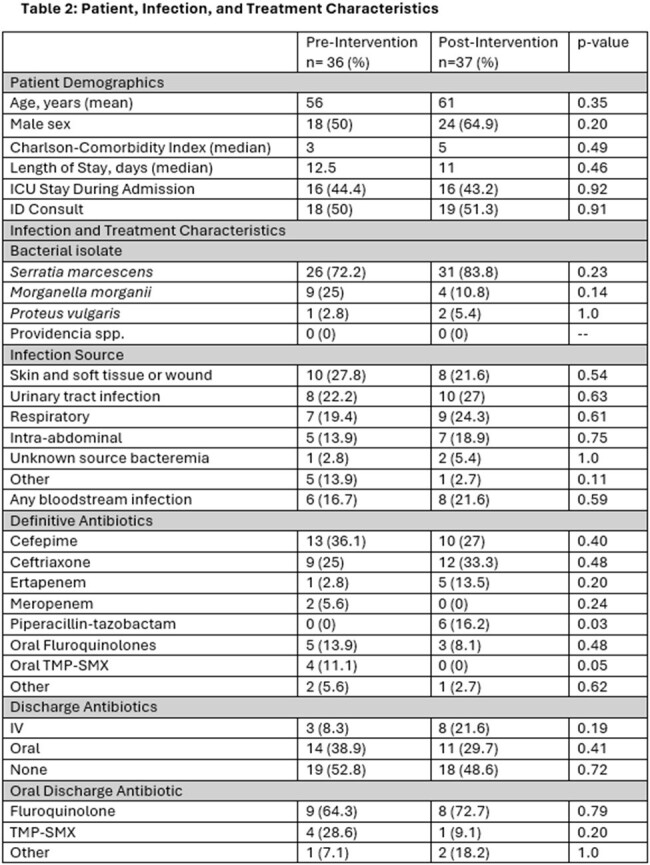

**Results:**

73 patients were included: 36 (49%) pre- and 37 (51%) post-intervention. Patient, infection, and treatment characteristics are described in **table 2.***Serratia marcescens* was the most common organism isolated with the most frequent sources being respiratory and urinary. There was no significant difference in the rate of carbapenem-sparing therapy between the pre- (83%) and post-(81%) intervention, p=0.59. No difference was found in CRO as definitive therapy in either group (25% vs. 33%, p=0.49) as well. No difference was seen in secondary clinical outcomes (**table 3**). For those with repeat positive cultures within 30 days, none developed CRO resistance.

**Figure:**
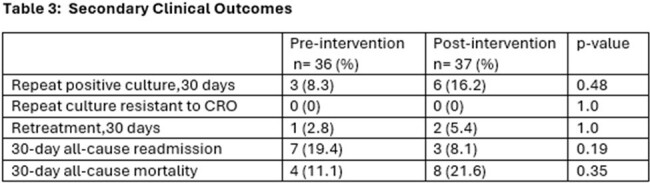

**Conclusion:**

The combination of changing microbiology reporting and a single ASP education session did not change antibiotic selection for low-risk AmpC producing organisms. Despite updated guidance the uptake in practice is slow.

**Disclosures:**

**All Authors**: No reported disclosures

